# Rheological Characteristics of Starch-Based Biodegradable Blends

**DOI:** 10.3390/polym15081953

**Published:** 2023-04-20

**Authors:** Dong-Il Park, Yuzhen Dong, Shizhao Wang, Soo-Jeong Lee, Hyoung Jin Choi

**Affiliations:** 1 R&F Chemical, Hanam 12925, Republic of Korea; rnf@rnfchemical.com (D.-I.P.); sjlee@rnfchemical.com (S.-J.L.); 2 School of Materials Science and Engineering, Harbin Institute of Technology at Weihai, Weihai 264209, China; yuzhend@hit.edu.cn; 3 Department of Polymer Science and Engineering, Inha University, Incheon 22212, Republic of Korea; 22201657@inha.edu

**Keywords:** starch, biodegradable, poly(butylene adipate-co-terephthalate), poly(lactic acid), melt rheology

## Abstract

Thermoplastic starch was blended with commercially available biodegradable polyesters of poly(butylene adipate-co-terephthalate) (PBAT) and poly(lactic acid) (PLA) for its improved performance and processability. The morphology and elemental composition of these biodegradable polymer blends were observed by scanning electron microscopy and energy dispersive X-ray spectroscopy, respectively, while their thermal properties were analyzed using thermogravimetric analysis and differential thermal calorimetry. For rheological analysis, the steady shear and dynamic oscillation tests of three samples at various temperatures were investigated using a rotational rheometer. All three samples exhibited significant shear thinning at all measured temperatures, and their shear viscosity behavior was plotted using the Carreau model. The frequency sweep tests showed that the thermoplastic starch sample exhibited a solid state at all temperatures tested, whereas both starch/PBAT and starch/PBAT/PLA blend samples exhibited viscoelastic liquid behavior after the melting temperature such that their loss modulus at low frequencies was greater than the storage modulus, and inversion occurred at high frequencies (storage modulus > loss modulus).

## 1. Introduction

With increasing attention given to global environmental issues, starch-based biodegradable polymer materials have attracted considerable attention as a substitute for substantial conventional petrochemical-based synthetic plastics. The development of biodegradable plastics has long been a major issue in the polymer community to reduce the impact of plastic waste on the environment. Starch is a natural biodegradable polymer that is abundant in plants such as corn, wheat, potato, and rice. In addition, starch is considered a promising candidate for developing biodegradable plastics because of its complete biodegradability, renewability, low cost, and low density [1,2]. On the other hand, its practical engineering application is quite limited because starch is sensitive to moisture and has relatively poor mechanical properties. Many studies have introduced various starch-based materials to meet their application requirements by blending them with other biodegradable polymers, such as polyvinyl alcohol [3], poly(lactic acid) (PLA) [4], polycaprolactone [5], poly(butylene succinate adipate) [6], poly(hydroxybutyrate-co-valerate) [7], and poly(butylene adipate-co-terephthalate) (PBAT) [8].

Among these, the PBAT is a biodegradable aliphatic–aromatic copolymer [9]. Starch/PBAT blends have been studied widely because of their interesting mechanical properties and barrier properties [10,11,12]. On the other hand, the incompatibility between hydrophilic starch and hydrophobic PBAT is usually known to lead to a decrease in mechanical properties of the starch/PBAT blends, such as the tensile strength, compressibility, resilience, and flexibility. Fourati et al. [13] investigated mechanical properties of the starch-based blend and starch/PBAT blend and pointed out that the breaking strain of pure starch-based blends was very low, and their modulus and tensile strength are very high. While the strain at break of the starch /PBAT blend increased notably to 185% compared with that of the starch-based blend, their physical properties related to rigidity decreased significantly. Therefore, adding an appropriate compatibilizer in the starch/PBAT blend system is necessary. Wei et al. [14] examined the performance of PBAT/potato starch blends by adding styrene-maleic anhydride-glycidyl methacrylate terpolymer as a compatibilizer or increasing the molecular weight of PBAT. The results showed that the mechanical properties are affected mainly by the starch content and dispersibility, and chain-extended PBAT could effectively improve the processing and mechanical properties.

Concurrently, PLA is a well-known biodegradable thermoplastic polyester that can be produced from agricultural products such as corn and sugar beets [15]. It is considered to be a promising alternative to traditional hydrocarbon-based plastics because of its excellent biodegradability, biocompatibility, high strength, high rigidity, and resistance to fats and oil [16,17,18]. On the other hand, pure PLA usually cannot be directly applicable owing to its brittleness, low thermal stability, low viscosity, and unsatisfactory gas barrier properties. The properties of PLA can be improved by blending with one or several biodegradable polymers, and this can integrate the advantages of each component and improve its performance and biodegradability. Teixeira et al. [19] conducted in-depth research on mechanical properties of starch/PLA blend and pointed out that although its elongation at break was reduced, compared with pure starch-based blend, its tensile strength, elastic modulus, and compressive strength were enhanced to some extent. Therefore, the combination of PBAT and PLA with their high toughness is considered an effective means to improve the material properties.

Moreover, various studies have been performed regarding their thermorheological properties. Using thermoplastic starch (TPS), Gonzalez et al. [20] showed that even though TPS did not obey the Cox–Merz relationship, its rheological properties were highly dependent on glycerol content. Meanwhile, for PBAT/PLA blend systems, Fourati et al. [13] reported that their viscoelasticity is jointly contributed by both PBAT and PLA, being related to the content ratio of the two. Shin et al. [21] also studied rheological characteristics of starch/PLA blend, in which the pure PLA showed Newtonian fluid with a power-law index around 0.95, while the blends showed strong pseudoplastic fluid with 0.65–0.11. The PBAT/PLA blend exhibited higher elongation at break than PLA, and the tensile strength can be increased by introducing fillers such as calcium carbonate (CaCO_3_) and carbon black [22,23]. Moreover, the blend of starch, PBAT, and PLA has also been studied, which can combine the advantages of these three components to adjust and improve mechanical properties, economic benefits, and biodegradability to meet different application requirements [24,25]. Furthermore, it is possible to expand the practical application field of biodegradable plastics using suitable compatibilizers and fillers and to adjust the ratio of each component.

In many polymer processing operations, such as extrusion, blow, and injection, the rheological properties are important for controlling processing conditions and the quality of the final product [26]. The melt rheological properties are of great significance for determining the processing and characteristics of polymer materials. For instance, associated with shear stress during processing of quinine blended with Eudragit^®^E100 (a cationic copolymer based on dimethylaminoethyl methacrylate, butyl methacrylate, and methyl methacrylate with a ratio of 2:1:1) and hydroxypropyl cellulose, the increased temperature reduces the shear viscosity of each polymer and its blends, implementing the process of pharmaceutical industry such as injection molding [27]. Moreover, in the case of asphalt with some viscoelastic properties, it was reported to be helpful to improve its resistance to rutting and fatigue cracking according to modification with adding waste ground tire rubber into asphalt to increase its shear viscosity and elastic modulus, even at high temperature, and reduce its energy storage and loss modulus at low temperature [28].

This study examined the steady shear and dynamic oscillatory rheological properties of starch-based biodegradable plastics, including starch and glycerin, starch and PBAT blend, and three-component blend with starch, PLA, and PBAT. Their surface morphologies were observed using scanning electron microscopy (SEM), and the chemical element composition was confirmed by energy dispersive X-ray spectroscopy (EDS). The thermal performance was analyzed using thermogravimetric analysis (TGA) and differential scanning calorimetry (DSC). Their melt rheological properties under various temperature conditions were studied using a rotational rheometer. Biodegradable ternary systems in this study could possess potential commercial applications in various areas, including agricultural mulching films, disposable plastic bags, disposable trays, disposable straws, and disposable plastic tableware. In addition, rheological data of the ternary blend system in this study would be beneficial for their compounding.

## 2. Experimental

### 2.1. Materials

Three commercial starch-based bioplastic materials called Tapioplast (S1), BioBlend AG202050 (S2), and Rodenburg compound (S3) were purchased from SMS Corporation (Lat Lum Kaeo, Thailand), BioLogiQ (Falls, ID, USA), and Rodenburg Biopolymers (Oosterhout, The Netherlands), respectively. Based on the information from each of the manufacturing companies, it was found that the composition of S1 is known to be more than 75% of tapioca starch and less than 25% of glycerol. S2 and S3 are blends of starch and biodegradable polymers. S2 contains mainly starch and PBAT with about 30% content of PBAT. S3 mainly contains starch, PBAT, and PLA at 16%, 18%, and 45%, respectively. In addition, some unknown additives might be added to improve their compatibilization or/and mechanical properties.

### 2.2. Characterization

The morphologies of the three samples were observed by SEM (HR-SEM, SU 8010, Hitachi, Japan), and their element compositions were examined using SEM-equipped EDS. The thermal characterizations, including thermogravimetric analysis (TGA), and differential scanning calorimetry (DSC), were performed using a thermal analysis system (STA 409 PC, DSC200F3, Netzsch, Germany). Their melt rheology was first tested by producing disc-shaped samples with a thickness of 1 mm and a diameter of 25 mm using a hot-pressing machine. The rheological properties at various temperatures were tested using a rotational rheometer (MCR 302, Anton Paar, Graz, Austria) with a parallel plate geometry (PP 25).

## 3. Results and Discussion

**Figure 1:**
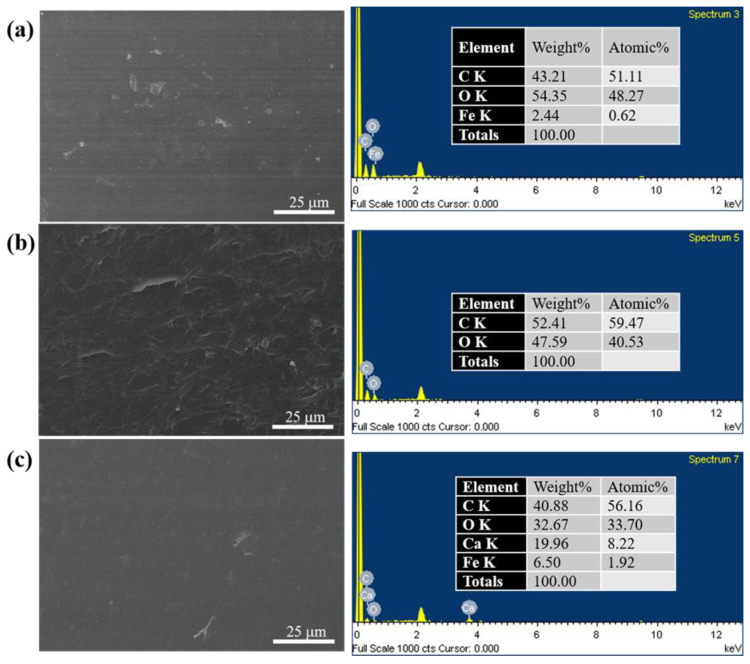
SEM images and EDS of (**a**) S1, (**b**) S2, and (**c**) S3.

Figure 1 presents the SEM images and EDS results of S1, S2, and S3. The surface of S1 is mostly smooth, and a small number of granules can be observed, i.e., presumably starch. For S2 of the starch/PBAT blend and S3 of the starch/PBAT/PLA blend, no apparent phase separation was observed in the SEM images, which means the compatibility between the components of these two blends is very high. The compatibility between hydrophilic starch and hydrophobic polyester is generally poor, so good phase compatibilization may be achieved by adding a certain compatibilizer. EDS showed that the main components of these three products are carbon and oxygen, and the small amount of iron detected in S1 and S3 is considered to be attached to the material during processing. The presence of calcium was detected in S3, so there are calcium-containing additives in S3.

**Figure 2:**
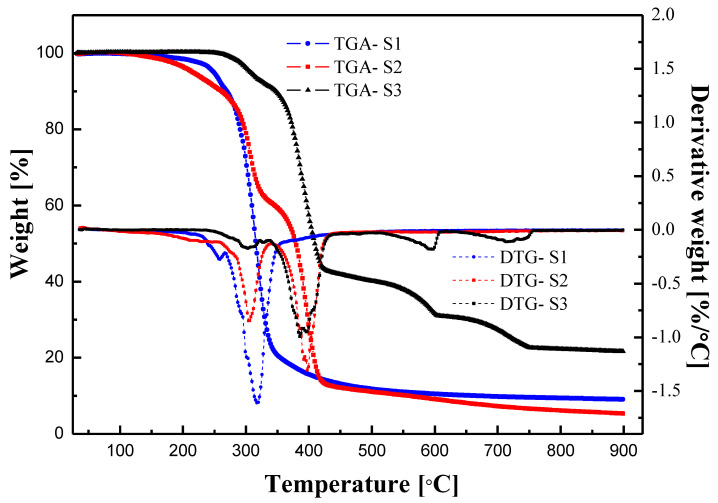
TGA and DTG curves of S1, S2, and S3.

Figure 2 shows the TGA and derivative thermogravimetric (DTG) curves of S1, S2, and S3 obtained by heating from 30 °C to 900 °C at a rate of 10 °C/min in a N_2_ atmosphere. The weight loss of S1, S2, and S3 occurs through two, three, and four steps, respectively. The weight lost before 190 ℃ was attributed to the evaporation of water. As shown in Table 1, S1 and S2 contain 1.3 wt.% and 2.8 wt.% water, respectively, while S3 contains almost no water. When the temperature exceeds 190 °C, the starch begins to decompose thermally, and the weight loss between 341 °C and 500 °C corresponds to the thermal decomposition of PBAT and PLA [22,29]. In addition, before 341 °C, the weight loss of S3 is only 9.1 wt.%, which means that the starch content in S3 is minimal. Furthermore, the two unexpected DTG peaks of S3 after 500 °C may be related to unknown additives. Combined with the EDS results, the DTG peak starting at 624 °C may be due to the thermal decomposition of calcium carbonate, while the peak at 500–624 °C may be due to the presence of calcium hydroxide [30,31,32].

**Figure 3:**
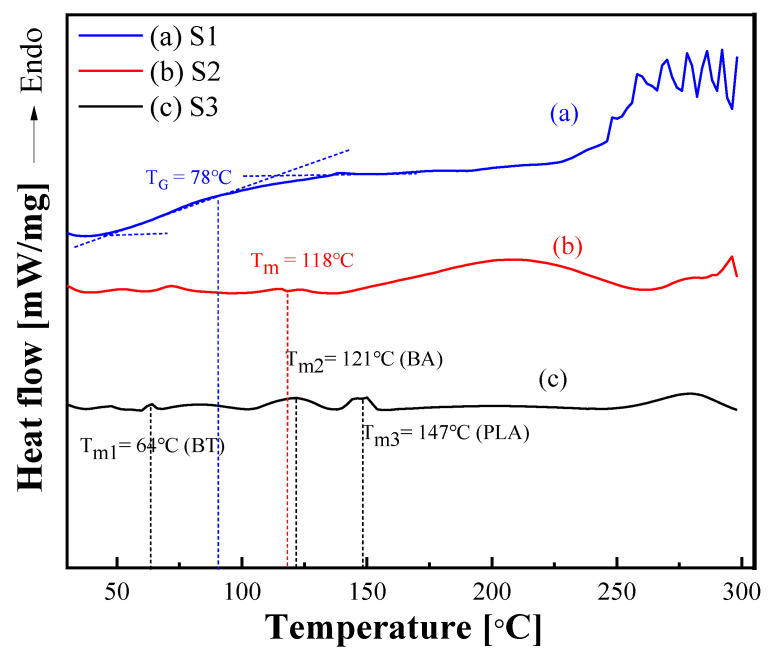
DSC curves of (**a**) S1, (**b**) S2, and (**c**) S3.

The DSC curves (Figure 3) of S1, S2, and S3 were analyzed by heating from 30 °C to 300 °C at a rate of 10 °C /min in a N_2_ atmosphere. The glass transition temperature (Tg) of S1 was approximately 78 °C, and no melting peak was observed. S2 showed a melting peak at 118 °C, which was attributed to the presence of PBAT, because PBAT is composed of two parts, butylene terephthalate (BT) and butylene adipate (BA), and their melting temperatures (Tm) were approximately 60 °C and 130 °C, respectively [33]. Therefore, the glass transition process of S2 was divided into two stages because of the endothermic melting effect of PBAT at approximately 60 °C. This exhibited a glass-transition-like step at 67 °C instead of a melting peak, probably because the PBAT content may be relatively small. S3 has three melting peaks, of which the peaks at 64 °C and 121 °C correspond to the melting process of the BT and BA segments of PBAT, respectively, while the peak at 147 °C was caused by the melting of PLA. In addition, S3 demonstrated a new glass transition process at around 55 °C due to the presence of PLA [34].

**Figure 4:**
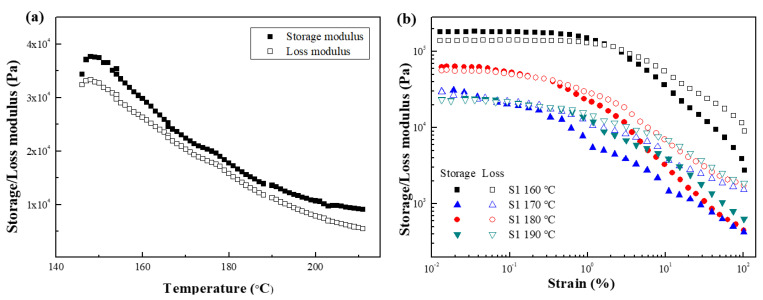
Storage (closed symbols) and loss (open symbols) modulus of (**a**) temperature sweep test and (**b**) strain amplitude sweep test for S1 at various temperatures.

The melt rheological properties of the samples were examined by first performing a temperature sweep test at a heating rate of 4 °C/min with a strain of 1% and an angular frequency of 6.28 rad/s. After checking melting temperature (Tm) by the temperature sweep test, the strain amplitude test and steady shear test were performed under various temperature conditions around the Tm temperature. Figure 4a presents the storage modulus (G′) and loss modulus (G″) of S1 in the temperature range of 140 °C to 220 °C, where G′ is always greater than G″, which proves that S1 is a solid-like state in the entire temperature range without melting. Figure 4b shows the G′ and G″ as a function of strain with a constant angular frequency of 6.28 rad/s at 160, 170, 180, and 190 °C. A linear viscoelastic (LVE) region appears in the low-strain area, and G′ > G″, demonstrating the solid-like state of S1. On the other hand, as the strain increases, the sample structure is destroyed, the modulus decreases rapidly, and a crossover point appears, after which G″ > G′. Moreover, the modulus decreases as the temperature is increased, which proves that the material gradually softens, and the sample begins to thermally degrade and no longer softens when the temperature is increased to 190 °C.

**Figure 5:**
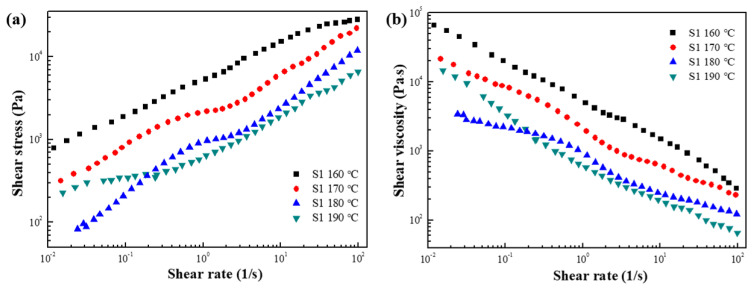
(**a**) Shear stress and (**b**) shear viscosity as a function of shear rate for S1 at various temperatures.

The shear stress and shear viscosity as a function of the shear rate under various temperatures were tested in controlled shear rate (CSR) mode. As shown in Figure 5a, in the range of 160–180 °C, the shear stress value gradually increases as the shear rate increases, and decreases as the temperature increases. The shear viscosity curve (Figure 5b) shows obvious shear thinning in a wide range of shear rates at all temperatures, and in the range of 160–180 °C, the shear viscosity also decreases as the temperature is increased. On the other hand, the shear stress and shear viscosity at 190 °C exhibits abnormal behavior, presumably due to the thermal decomposition of the sample due to excessive temperature.

**Figure 6:**
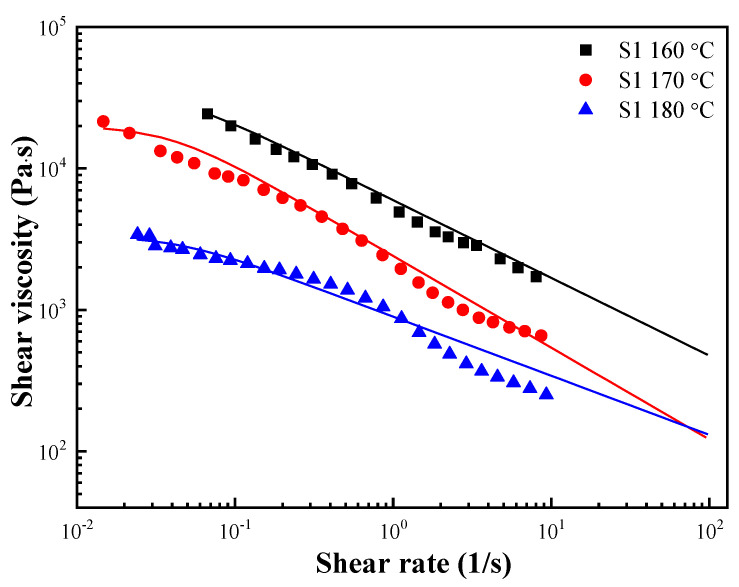
Shear viscosity as a function of the shear rate for S1 at various temperatures. The solid lines were fitted using the Carreau model.

In addition, various models have been developed to measure shear viscosity at high shear rates owing to the difficulty in practical measurements [35]. The Carreau model is the most commonly used model, and can be expressed as follows [36]:

(1) $$\eta =\frac{\eta_{0}}{{\left[1+\lambda {\left(\dot{\gamma }\right)}^{2}\right]}^{\frac{\left(1-n\right)}{2}}}$$

where λ is the relaxation time; $\eta_{0}$ is the zero-shear viscosity; n is the dimensionless parameter and can describe the flow state. When 0 ≤ *n* < 1 and *n* = 1, it indicates Newtonian flow. The solid lines in Figure 6 show the fitting result of the Carreau model, and Table 2 lists the fitting parameters at each temperature.

**Figure 7:**
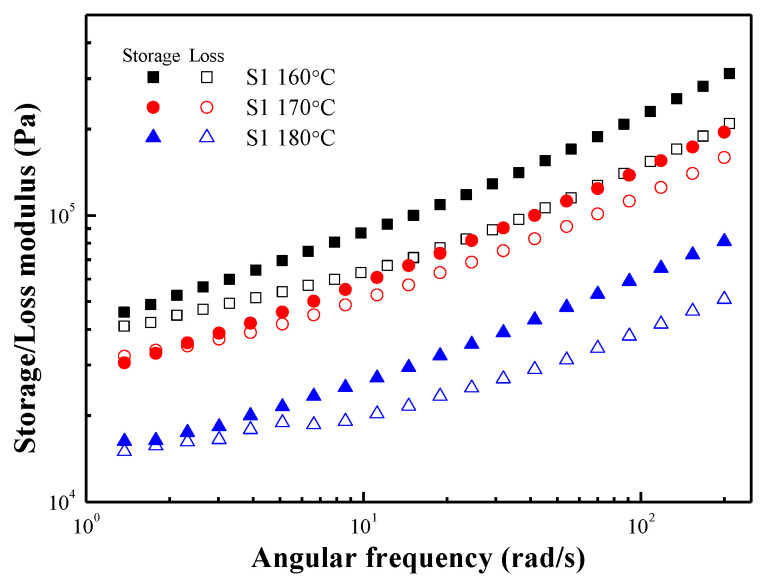
Frequency-dependent storage modulus (closed symbols) and loss modulus (open symbols) of S1 at various temperatures.

The dependence of the material on frequency was examined by performing the frequency sweep test at a fixed strain of 0.01% for each temperature. As shown in Figure 7, G′ and G″ increased as the frequency increased and decreased as the temperature increased. On the other hand, at 160, 170, and 180 °C, the G′ was higher than the G″ in the entire frequency range and exhibited a solid-like behavior, indicating that the S1 was not melted.

**Figure 8:**
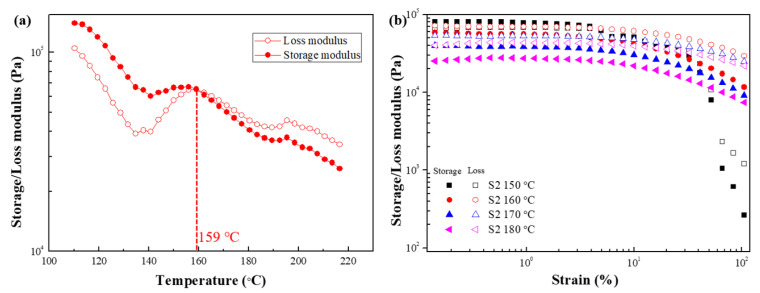
Storage (closed symbols) and loss (open symbols) modulus of (**a**) temperature sweep test and (**b**) strain amplitude sweep test for S2.

Figure 8a shows the temperature sweep test result of S2 at 110–220 °C. The intersection of G′ and G″ appeared at 159 °C, after which the G″ > G′, meaning that the melting temperature of S2 is 159 °C. The strain amplitude test was carried out in the temperature range of 150–180 °C to compare the changes in the rheological properties of the sample before and after the melting temperature and the influence of temperature on the rheological properties (Figure 8b). Before reaching the melting temperature (150 °C), G′ > G″ and exhibits solid-like properties, whereas when the temperature exceeds the melting temperature (160 °C, 170 °C, and 180 °C), G″ > G′, indicating liquid-like properties. In addition, the modulus decreases as the temperature increases, which means that the sample adopts a liquid-like state at higher temperatures.

**Figure 9:**
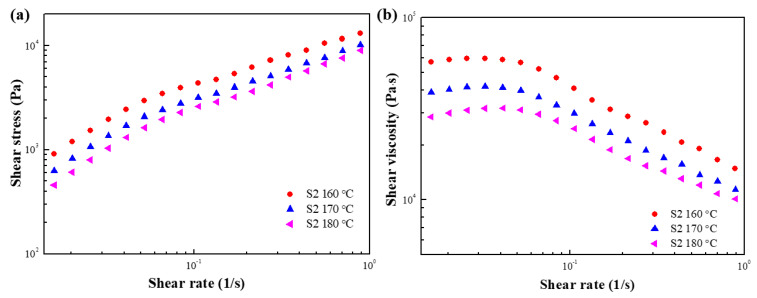
(**a**) Shear stress and (**b**) shear viscosity as a function of shear rate for S2 at various temperatures.

**Figure 10:**
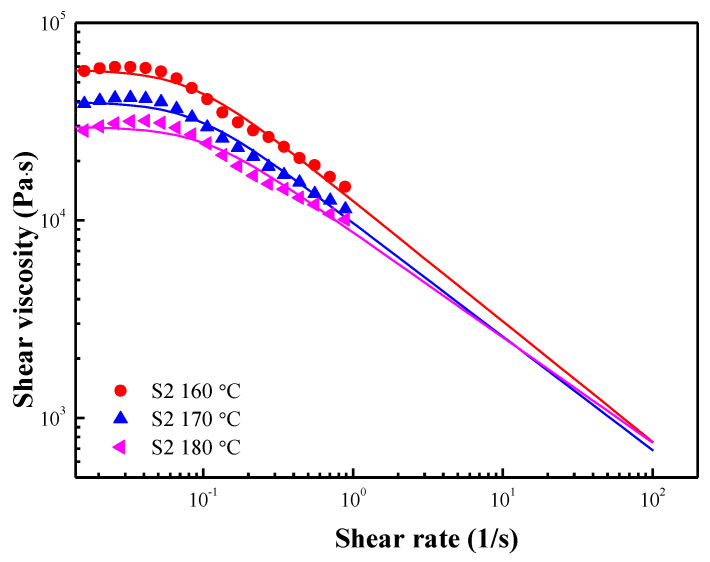
Shear viscosity as a function of shear rate for S2 at various temperatures. The solid lines were fitted by the Carreau model.

Figure 9 shows the shear stress and shear viscosity curves of S2 at 160, 170, and 180 °C. At all temperatures, the shear stress increases gradually as the shear rate increases while undergoing significant shear thinning. Moreover, the shear stress and shear viscosity increase as the temperature increases. Compared with S1, the increase of shear stress and shear viscosity for S2 is thought to be due to the friction between the macromolecular chains in PBAT [37]. The shear viscosity behavior at a high shear rate was fitted by the Carreau model (Equation (1)), as shown in Figure 10, and Table 3 shows the fitting parameters.

**Figure 11:**
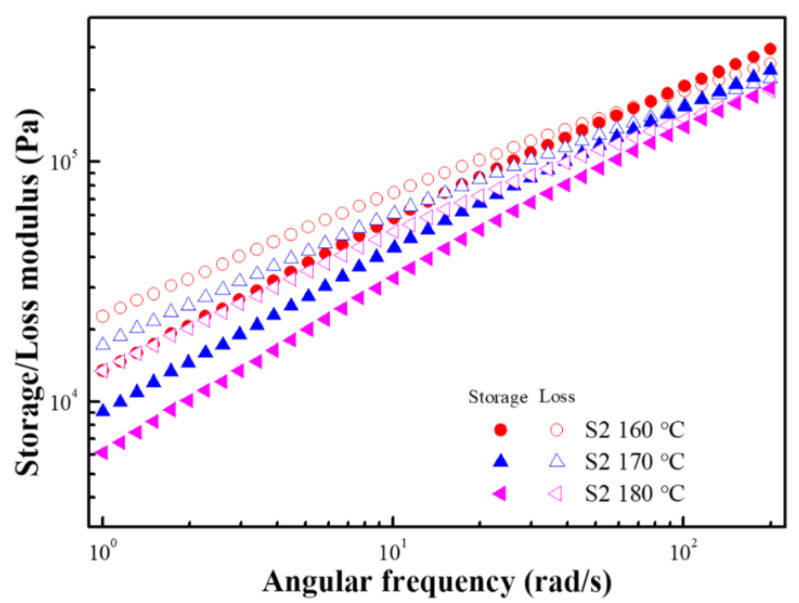
Frequency-dependent storage modulus (closed symbols) and loss modulus (open symbols) of S2 at various temperatures.

G′ and G″ are not constants but change with time because viscoelastic materials are time-dependent. In the dynamic oscillation tests, the time dependence can be evaluated by changing the frequency of the applied stress or strain. Figure 11 shows the G′ and G″ of S2 over the frequency range of 1 to 200 rad/s, where the dynamic moduli decrease as the temperature increases and exhibit viscoelastic liquid behavior. In the case of Maxwell’s model, the G′ and G″ dependence on frequency can be expressed using the following formulae [38]:

(2) $$G^{\prime }=\frac{G{\left(\omega \lambda \right)}^{2}}{1+{\left(\omega \lambda \right)}^{2}}$$

(3) $$G^{\prime \prime }=\frac{}{1+{\left(\omega \lambda \right)}^{2}}$$

where λ represents the relaxation time, which can be regarded as the reciprocal of the frequency at the intersection of the G′ and G″ curves. As shown in Figure 11, G′ and G″ intersect at high frequency, which is related to the relaxation of the material. In the high-frequency region (short time), the storage modulus is greater than the loss modulus and exhibits solid-like behavior. In the low-frequency region (long time), the loss modulus is greater than the storage modulus and exhibits liquid-like behavior. This result is because the stored elastic stress relaxes through the rearrangement of the microstructure and transforms into viscous stress. In addition, as the temperature increases, the frequency of the intersection increases, showing that the relaxation time of the material decreases as the temperature increases.

**Figure 12:**
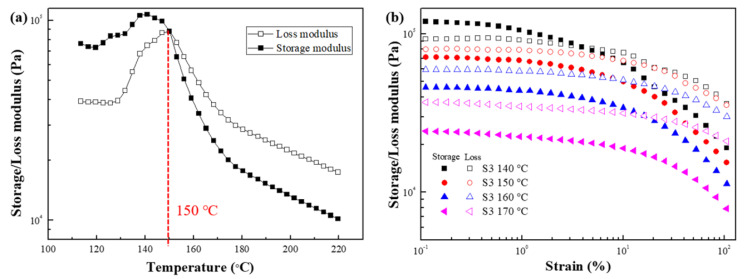
Storage (closed symbols) and loss (open symbols) modulus of (**a**) temperature sweep test and (**b**) strain amplitude sweep test for S3.

Figure 12a shows the temperature sweep test result of S3 in the temperature range of 110–220 °C. The intersection of G′ and G″ appears at 150 °C, indicating that the melting temperature of S3 is 150 °C, which is lower than that of S2. The strain amplitude test (Figure 12b) was performed between 140 °C and 170 °C to confirm the change in the rheological properties of the sample before and after the melting temperature and the effect of temperature on the rheological properties. At 140 °C, the temperature was lower than the melting temperature, so G′ was significantly greater than the G″ in the lower-strain region, indicating that the sample was in a solid-like state. On the other hand, when the temperature was higher than the melting temperature, the G″ > G′ over the entire strain range, and the sample became liquid-like. The liquid properties became more evident as the temperature increased.

**Figure 13:**
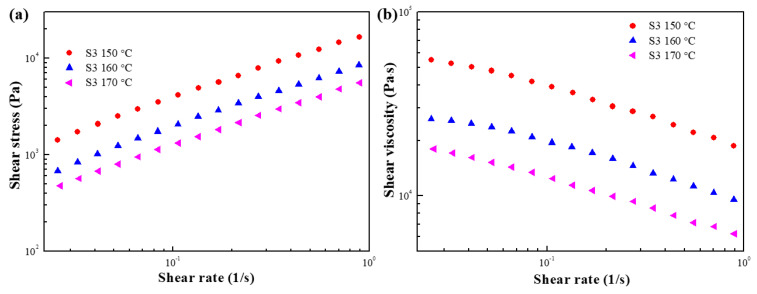
(**a**) Shear stress and (**b**) shear viscosity as a function of the shear rate for S3 at various temperatures.

**Figure 14:**
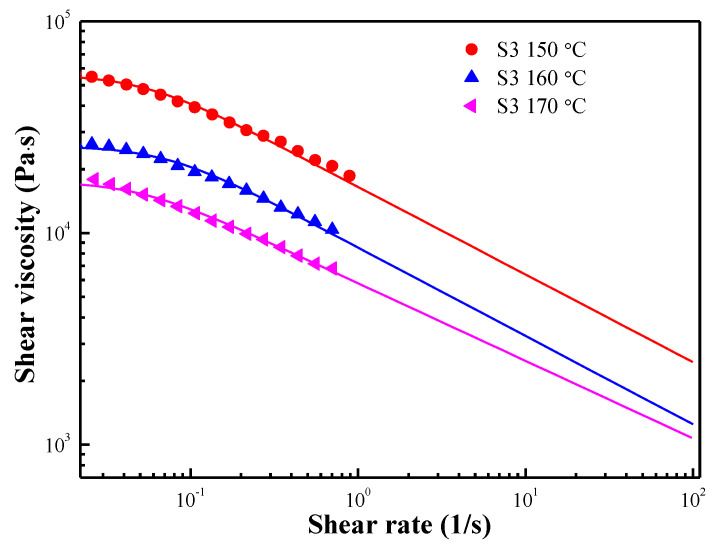
Shear viscosity as a function of the shear rate for S3 at various temperatures. The solid lines are fitted by the Carreau model.

Figure 13a,b show the shear stress and shear viscosity of S2 as a function of the shear rate at 150, 160, and 170 °C. At all temperatures, the shear stress increased gradually as the shear rate increased, and the shear viscosity exhibited significant shear thinning behavior. Moreover, the shear stress and shear viscosity decreased as the temperature increased. As described above, because it is difficult to measure shear viscosity at high shear rates, the shear viscosity behavior was predicted through the Carreau model (Equation (1)). Figure 14 and Table 4 show the prediction results and fitting parameters for S3, respectively. Furthermore, the shear viscosity of S3 was lower than that of S1 and S2 because of the lowest starch content. In addition, the decrease in shear viscosity of S2 and S3 might correspond to a loss in molecular weight according to the related literature [39].

**Figure 15:**
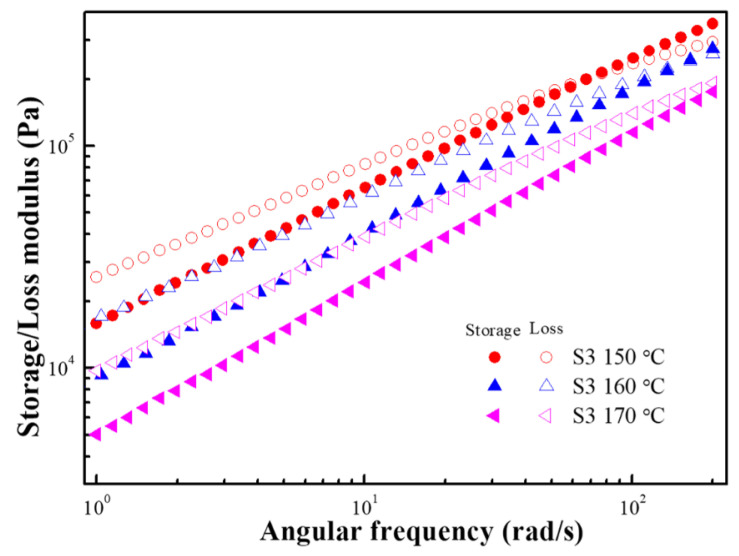
Frequency-dependent storage modulus (closed symbols) and loss modulus (open symbols) of S3 at various temperatures.

Figure 15 shows G′ and G″ as a function of the angular frequency at 150 °C, 160 °C, and 170 °C. G′ and G″ increased as the frequency increased and decreased as the temperature increased. At 150 °C and 160 °C, G″ > G′ in the low-frequency region, showing liquid-like behavior, and an intersection point appeared in the high-frequency region. By contrast, at 170 °C, G″ > G′ in the entire frequency range, and no intersection point was observed, which is expected to occur at a higher frequency. Therefore, the frequency of the intersection points increased as the temperature increased, i.e., the relaxation time became shorter as the temperature was increased.

## 4. Conclusions

Three starch-based biodegradable materials of starch, starch/PBAT blend, and starch/PBAT/PLA blend were characterized using SEM, EDS, TGA, and DSC. The melt rheological properties, including steady shear and dynamic oscillation tests at various temperatures, were investigated using a rotational rheometer. SEM revealed the two blend systems of S2 and S3 to have good phase compatibility. Using EDS and TGA, it was speculated that there were calcium-containing additives in S3, and the content of starch in S3 was relatively small. S1 showed a wide glass transition region, and no melting peak was observed. Affected by the melting of PBAT, the glass transition process of S2 was divided into two stages, and a melting peak appeared at 118 °C. Owing to the presence of PLA, the glass transition process of S3 appeared in two stages, and there were three melting peaks. In the melt rheological analysis, S1 failed to reach the molten state until 220 °C, and the melting temperatures of S2 and S3 were 159 °C and 150 °C, respectively. The storage modulus, loss modulus, shear stress, and shear viscosity decreased with increasing temperature. All samples exhibited significant shear thinning at all temperatures, and the shear viscosity behavior of all products at a high shear rate can be predicted by the Carreau model. The frequency sweep tests showed that S1 exhibited a solid state at all tested temperatures, while S2 and S3 exhibited viscoelastic liquid behavior after the melting temperature. In addition, in the case of S2 and S3, the loss modulus at low frequencies was greater than the storage modulus, and inversion occurred at high frequencies (storage modulus > loss modulus). Furthermore, the frequency of intersection became higher at higher temperatures, which means that the relaxation time became shorter. Therefore, considering these thermorheological properties within high range of temperature, S1, which showed solid-like properties at high temperature, can be applied in high-temperature thermo-processing, while due to its low Tm, it is easier to carry out the molding process at a relatively low temperature during processing.

## Figures and Tables

**Table 1:** Thermal properties of S1, S2, and S3.

Materials		Step 1	Step 2	Step 3	Step 4
**S1**	Temperature range (°C)	~190	190–900		
	Weight loss (wt.%)	1.3	89.6		
**S2**	Temperature range (°C)	~190	190–341	341–900	
	Weight loss (wt.%)	2.8	36.6	55.2	
**S3**	Temperature range (°C)	254–341	341–500	500–624	624–900
	Weight loss (wt.%)	9.1	50.8	9.4	9.0

**Table 2:** Fitting parameters of the Carreau model for S1 under various temperatures.

Materials	$\eta_{0}$ (Pa·s)	λ (s)	n	$R^{2}$
S1 160 °C	3.56 × 10^5^	658	0.43	0.97
S1 170 °C	2.01 × 10^5^	688	0.35	0.98
S1 180 °C	3.32 × 10^4^	527	0.58	0.97

**Table 3:** Fitting parameters of the Carreau model for S2 under various temperatures.

Materials	$\eta_{0}$ (Pa·s)	λ (s)	n	$R^{2}$
S2 160 °C	5.79 × 10^5^	152	0.39	0.96
S2 170 °C	3.98 × 10^5^	135	0.43	0.95
S2 180 °C	2.97 × 10^4^	101	0.47	0.93

**Table 4:** Fitting parameters of the Carreau model for S3 under various temperatures.

Materials	$\eta_{0}$ (Pa·s)	λ (s)	n	$R^{2}$
S3 150 °C	5.61 × 10^5^	367	0.59	0.93
S3 160 °C	2.58 × 10^5^	197	0.58	0.90
S3 170 °C	1.75 × 10^4^	422	0.63	0.94

## Data Availability

The data that support the findings of this work are available from the corresponding author upon reasonable request.
